# Which muscles exhibit increased stiffness in people with chronic neck pain? A systematic review with meta-analysis

**DOI:** 10.3389/fspor.2023.1172514

**Published:** 2023-08-30

**Authors:** Manca Opara, Žiga Kozinc

**Affiliations:** ^1^ Faculty of Health Sciences, University of Primorska, Izola, Slovenia; ^2^ Department of Health Studies, Andrej Marušič Institute, University of Primorska, Koper, Slovenia

**Keywords:** chronic neck pain, shear modulus, elastography, myotonometry, upper trapezius

## Abstract

**Introduction:**

Chronic neck pain (CNP) substantially impacts quality of life, posing both personal challenges and economic strains. This systematic review sought to discern muscle-specific stiffness differences between CNP patients and individuals without CNP.

**Methods:**

We searched the PubMed, Scopus, and PEDro databases for studies using ultrasound elastography or myotonometry to compare muscle stiffness between CNP patients and asymptomatic controls. Using a meta-analysis with a random-effects model, we derived the pooled effect as standardized mean difference (SMD).

**Results:**

Out of the six studies selected, the adjusted Newcastle-Ottawa rating scale for cross-sectional studies denoted three as moderate-quality and three as high-quality. Our findings indicate that the upper trapezius (UT) stiffness was elevated in CNP patients compared to their counterparts without CNP (SMD = 0.39, 95% CI = 0.05 to 0.74; *p* = 0.03; small effect size). The data for other muscles remained inconclusive.

**Discussion:**

Given the case-control design of all reviewed studies, a direct causative link between UT stiffness and CNP is yet to be confirmed. As such, recommending a reduction in trapezius muscle stiffness as a primary rehabilitation strategy for CNP patients is still inconclusive and further research is needed.

## 1. Introduction

Neck pain (NP), ranking as the fourth major cause of disability, adversely impacts quality of life, leading to dissatisfaction at work, challenges in daily activities, and substantial personal and economic implications (1–3). With an annual prevalence rate of 37.2%, NP affects up to 67% of individuals at some point in their lives (4, 5). NP lasting over three months is termed chronic NP (CNP) (6). People suffering from CNP may experience restlessness, visual disturbances, neuromuscular dysfunction, and limited mobility of the cervical spine (7–10). Pain experiences can prompt individuals to rely less on superficial neck and back muscles, leading to weakening of deeper muscles (11). Such neuromuscular adaptations and muscular property shifts in NP patients are often associated with complaints of tension, tightness, or stiffness in neck muscles (12, 13). Previous studies reported altered muscle cross-sectional area, thickness, size, and activity of deep neck muscles, decreased neck muscle strength, and existing myofascial trigger points in NP patients (14–17). An increase in neck muscle stiffness can result in muscle dysfunction and excess strain on the cervical spine, intensifying pain sensations and potentially fueling a persistent pain cycle (18, 19).

While CNP patients often subjectively report increased neck stiffness (13, 20), objective measures of muscle stiffness can offer deeper insights into CNP's underlying mechanisms and guide therapeutic interventions. Ingram et al. (19) identified heightened joint stiffness at the cervical spine in those with nonspecific neck pain. Yet, this stiffness was not directly linked to pain severity or disability levels. A emerging area of research is the assessment of muscle-specific stiffness through ultrasound elastography (21, 22). Elastography is based on stress application and tracking of tissue displacement (23). It entails generating an acoustic or mechanical wave and subsequently tracking its propagation speed; higher speeds indicate stiffer tissues (24, 25). Ultrasound elastography has proven particularly reliable in assessing stiffness in neck muscles such as the trapezius (26, 27), sternocleidomastoid (28) and deep cervical extensors (29). Another method that allows the assessment of muscle-specific stiffness is myotonometry, which is based on tracking the mechanical oscillations after a mechanical impulse (30). However, muscle stiffness results obtained with myotonometers do not always agree with elastography-based stiffness (31, 32). This discrepancy might stem from shear-wave elastography's ability to scrutinize specific regions of interest, while myotonometry largely captures superficial tissues and is influenced by factors like skin thickness and body fat percentage. Nonetheless, owing to its affordability, ease of use, and high reliability, myotonometry is gaining attraction as a valuable clinical tool in assessing muscle stiffness (30, 33).

Recent research indicates heightened stiffness in several neck muscles among CNP patients. For example, Taş et al. (21) found increased stiffness in the upper trapezius, levator scapulae, and sternocleidomastoid (but not the splenius capitis) in CNP patients compared to those without symptoms. While other studies have similarly highlighted elevated stiffness in the trapezius muscle of CNP patients (34, 35), there is no documented increased stiffness in muscles such as the multifidus and semispinalis cervicis (36). Although muscle stiffness may not be related to pain symptoms and degree of disability (19, 21, 36), muscle-specific assessments of stiffness could be used to guide rehabilitation programs. Muscles displaying increased stiffness might be prioritized for stretching interventions, while those with reduced stiffness may benefit more from strengthening exercises. To date, a comprehensive systematic review on muscle-specific stiffness variations in CNP patients remains absent. Hence, our systematic review, complemented with a meta-analysis, aims to discern which muscles show elevated stiffness in those suffering from CNP.

## 2. Methods

A systematic review was performed to identify randomized clinical trials exploring the differences in muscle stiffness between patients with CNP and asymptomatic control groups. This review was executed in line with the guidelines established by the Preferred Reporting Items for Systematic Reviews and Meta-Analyses (PRISMA) (37).

### 2.1. Eligibility criteria

We considered case-control cross-sectional studies that compared muscle stiffness between idiopathic CNP patients with pain lasting >3 months and a matched asymptomatic control group. Experimental studies involving both CNP patients and an asymptomatic control group were also considered, with only baseline data being retrieved. Studies were excluded if participants suffered from specific pathologies associated with CNP, such as migraines or myofascial syndrome. Regarding the muscle stiffness outcomes, we considered ultrasound elastography and myotonomtery. Segment or joint-level stiffness assessments (e.g., passive resistance to externally-induced neck movement) were not considered, as our research question was focused on stiffness changes in specific muscles. Aiming to offer a comprehensive review, we incorporated both passive (relaxed muscle) and active (contracted muscle) stiffness outcomes. We then performed subgroup and sensitivity analyses to discern if the differences between CNP patients and the control group were contingent upon the stiffness measurement type. Inclusion criteria included original articles, either full-length or brief communications, published in peer-reviewed journals. While our database search didn't restrict based on language or publication date, only English articles were selected for data extraction.

### 2.2. Information sources and selection process

**Figure 1:**
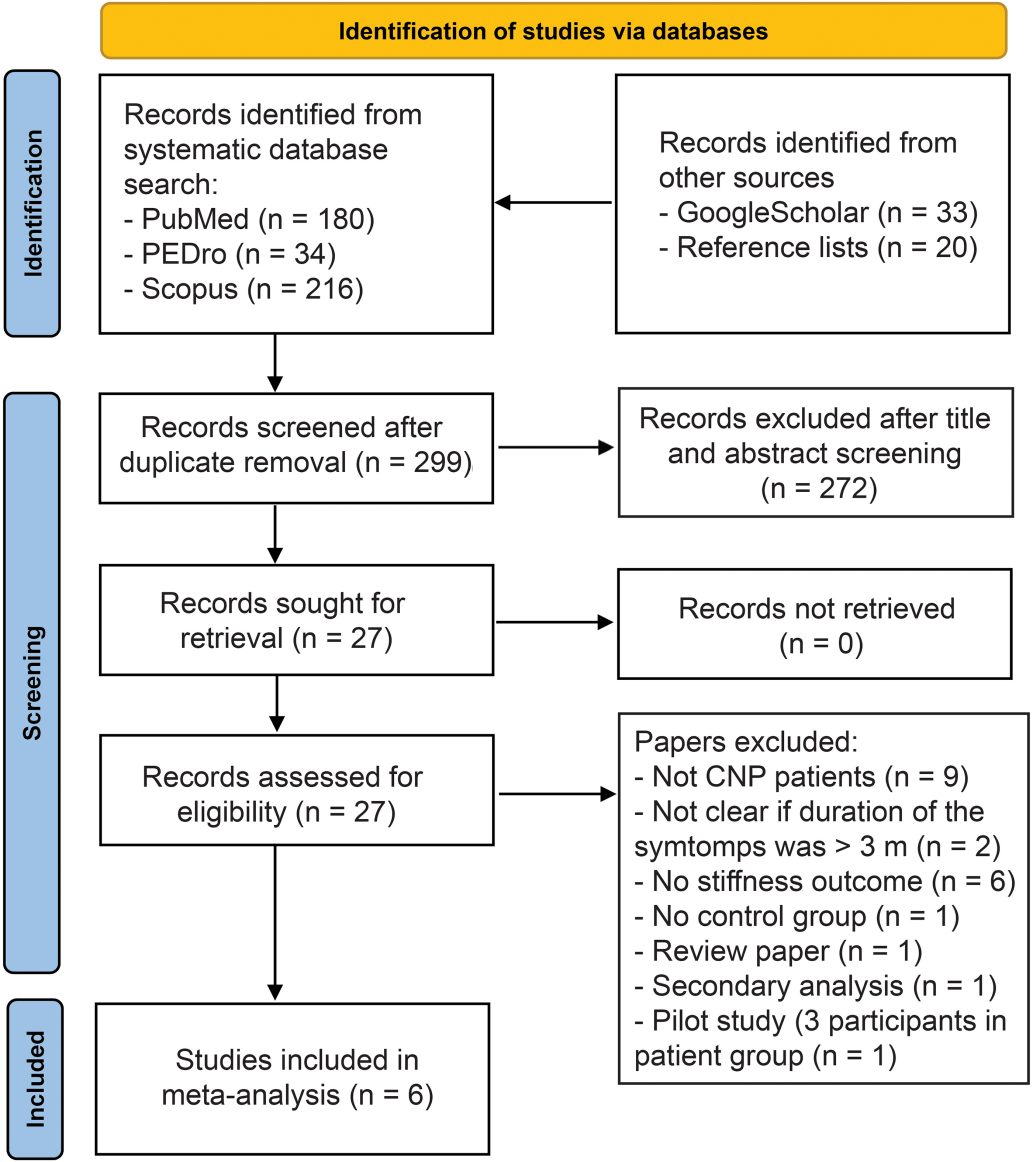
Overview of article search and study selection.

The search was performed in January 2023 and revised in August 2023. Both authors independently conducted all steps of the review. Potential disagreements were resolved by discussion. We searched the PubMed, Scopus and PEDro databases. In MeSH terms database, “neck pain”, “muscle tone”, and “elastography” were identified. To ensure a comprehensive search, further synonyms were added. For PubMed and Scopus, we used the following search string: (“*neck pain*” *OR* “*neck syndrome*” *OR* “*cervical pain*”) *AND* (*muscle stiffness OR muscle tone OR elastography OR myotonometry*)*.* During the manuscript revision stage (August 2023), additional MeSH terms “muscle tonus” and “Elasticity Imaging Techniques” were added to the search string, with no additional papers identified. In the PEDro database, a simple search was used with individual combinations of words, such as “neck pain stiffness”, “muscle stiffness neck” “elastography neck pain” and “myotonometry neck pain” were used. While Scoups and PEDro databases were also explored for studies that used terminology similar to the specified MeSH terms, it has to be recognized that they do not utilize PubMed's MeSH terms or subject headings. In addition, we (1) reviewed the reference lists of identified systematic reviews on the topic (2) reviewed the reference lists papers already obtained through the abovementioned databased (3) performed an additional non-systematic search of the Google Scholar search engine with the same key words as used in PEDro database. The records were imported into Mendeley (version 1.19.8) to remove the duplicates, and then exported into Microsoft Excel software for further steps. After the duplicates were removed, the authors examined the titles and abstracts, and excluded the studies that obviously did not fit the research question. In case there was a disagreement between the authors, the paper was carried over to the next stage. Then, a full-text examination was performed, and data extraction was performed for the articles that met the exclusion criteria. The reasons for exclusions were noted and listed in the PRISMA flowchart (Figure 1).

### 2.3. Data collection and data items

Both authors independently undertook the data extraction process. Any discrepancies encountered were settled through meticulous review and discussion. The data collated encompassed: (a) muscle stiffness figures for CNP and control groups, including means and standard deviations; (b) demographic details such as gender, age, height, weight, and body mass index; (c) specifics of the measurement processes, including the device used, type and units of the outcome measure, measurement location, and participant's posture.

All data were organized using Microsoft Excel 2016 (Microsoft, Redmond, WA, USA). When data was solely presented graphically, we utilized the GetGraphDataDigitalizer software (version 2.26.0.20) to extract the means and standard deviations. For any instances of incomplete data, we reached out to the respective article authors via email and ResearchGate. If there was no communication 14 days post the second reminder, we deemed the data as irretrievable.

### 2.4. Assessment of study quality

We employed the Newcastle–Ottawa quality assessment scale for Cross-Sectional studies (38) to evaluate the quality of the included studies. To align more closely with our research question, we made slight modifications to the scale. The “respondents” item was omitted, and “ascertainment of exposure” was substituted with “patient criteria.” A study met this criterion if it clearly outlined the inclusion parameters (such as symptom duration and nature) and exclusion factors (like the presence of specific pathologies) for the CNP groups. The adapted scale featured six items, classifying studies as high quality (score of 6), moderate quality (scores of 4–5), low quality (scores of 2–3), and poor quality (scores of 0–1).

### 2.5. Effect measures and synthesis methods

The meta-analysis was conducted in Review Manager (Version 5.3, Copenhagen: The Nordic Cochrane Center, The Cochrane Collaboration, London, UK). There is no consensus on the minimal number of studies required for a meta-analysis (39), therefore, we performed the analyses when two or more studies could be included for each muscle. We used continuous outcomes meta-analysis with a random-effects model and inverse variance method to obtained the pooled difference between CNP and control groups. The effect sizes were expressed as standardized mean difference (SMD) with 95% confidence intervals (CI), as the units of measurement were not consistent across studies. Statistical heterogeneity among studies was assessed with the *I*^2^ statistics, following the Cochrane guidelines (the *I*^2^ statistics of 0%–30% might not be important, 30%–60% may represent moderate heterogeneity, 60%–90% may represent substantial heterogeneity, and 90%–100% indicates considerable heterogeneity). The threshold for statistical significance was set at *p* ≤ 0.05 for the pooled effect size. In case of substantial heterogeneity between the studies, a sensitivity analysis was performed by examining the effect of exclusion of studies that differed significantly from the pooled effect.

## 3. Results

### 3.1. Study selection

The literature search is summarized in Figure 1, and the basic details for each study is presented in Table 1. We included 6 studies into the meta-analysis (Table 1).

**Table 1:** Characteristics of included studies.

Authors	Participants			Pain symptoms	Stiffness outcome	Muscles included and position during testing
	Numerus	Age in years (mean ± SD)	Sex distribution (M/F)			
Dieterich et al. (36)	*n* = 38 CNP = 20 C = 18	CNP = 52.5 ± 12.0 C = 48.5 ± 9.0	Only women.	>6 months	Shear modulus (kPa)	U. trapezius, splenius capitis, semispinalis capitis/cervicis, multifidus (all P)
Heredia-Rizo et al. (41)	*n* = 40 CNP = 20 C = 20	CNP = 46.7 ± 6.1 C = 23–67 years	Only women.	>3 months	Myotonometry stiffness (N/m)	U. trapezius (P)
Ishikawa et al. (22)	*n* = 30 CNP = 18 C = 12	CNP = 25.7 ± 2.9 C = 24.5 ± 3.9	P = 9/9 C = l 8/4	Neck and shoulder complaints for >3 months in the past year, at least once in the past week and on the day of the measurement procedures.	Strain ratio	U. trapezius (SIT), levator scapulae (SIT), rhomboid major (SIT)
Taş et al. (21)	*n* = 70 CNP = 35 C = 35	CNP = 35.6 ± 8.3 C = 35.2 ± 9.2	P = 8/27 C = 11/24	>3 months	Shear-wave velocity (m/s)	U. trapezius (P), levator scapulae (P), SCM (S), splenius capitis (P)
Wolff et al. (40)	*n* = 36 CNP = 18 C = 18	CNP = 34.4 ± 2.5 C = 32.3 ± 3.0	P = 7/11 C = 7/11	>3 months	Shear-wave velocity (m/s)	U. trapezius (P), SCM (S)
Xie et al. (42)	*n* = 49 CNP = 18 C = 31	CNP = 39.3 ± 11.5 C = 38.8 ± 10.8	P = 3/15 C = 5/26	Patients with mild, moderate and severe neck disability	Shear modulus (kPa)	Upper/middle/lower/posterior/anterior trapezius, spinalis capitis, semispinalis capitis/cervicis, multifidus, levator scapulae, serratus anterior (all SIT)

CNP, chornic neck pain group; C, control group; S, supine; P, prone; SIT, seated.

### 3.2. Study characteristics

The muscles represented were upper trapezius (6 studies), levator scapulae (3 studies), sternocleidomastoideous (2 studies), splenis capitis (2 studies), multifidus (2 studies), semispinalis capitis (1 study), rhomboid major (1 study) and semispinalis cervicis (1 study). All studies included adult population (range of means for age: 24.5–52.5 years). Most studies (5/6) reported body mass index or a combination of body height and body mass data, with all groups means fitting into the normal weight category (range of means for body mass index: 20.6–24.9). Most studies assessed passive muscles stiffness, with participants lying prone our supine on an examination table, except for Wolff et al. (40), who investigated muscle stiffness during functional reach tasks. Two studies (36, 41) included only women and the rest included both men and women. Four studies reported neck disability index scores that ranged from 15.1 ± 1.3 points (40) to 32.5 ± 12.3 points (36). Four studies also reported mean pain using VAS or NRS scales, with scores ranging from 2.4 ± 1.6 (22) to 4.6 ± 1.9 (21). Inclusion criteria for CNP group differed substantially across studies; for instance, three studies included participants with CNP lasting >3 months (21, 40, 41), while >6 months (36) have also been used. To assess muscle stiffness, one study used myotonometry (41) and the other five used ultrasound elastography. The studies were somewhat heterogenous regarding participant positioning and measurement locations. The details concerning participant characteristics, methodology of each study and extracted outcome data are available in Supplementary File S1.

### 3.3. Assessment of study quality

The studies we included were categorized as either high quality (3 studies) or moderate quality (3 studies). A notable omission across these studies was the calculation of sample size; only half of them (3 out of 6) furnished a comprehensive description. Detailed information can be found in Table 2.

**Table 2:** Summary of study quality assessment.

Study	Representativeness of the sample	Sample-size	Patient criteria defined	Comparability of groups	Assessment of the outcome	Statistical testing	Total
Dieterich et al. (36)	1	0	1	1	1	1	5
Heredia-Rizo et al. (41)	1	1	1	1	1	1	6
Ishikawa et al. (22)	1	0	1	1	1	1	5
Taş et al. (21)	1	1	1	1	1	1	6
Xie et al. (42)	1	0	0	1	1	1	4
Wolff et al. (40)	1	1	1	1	1	1	6

### 3.4. Differences between CNP patients and control groups

**Figure 2:**
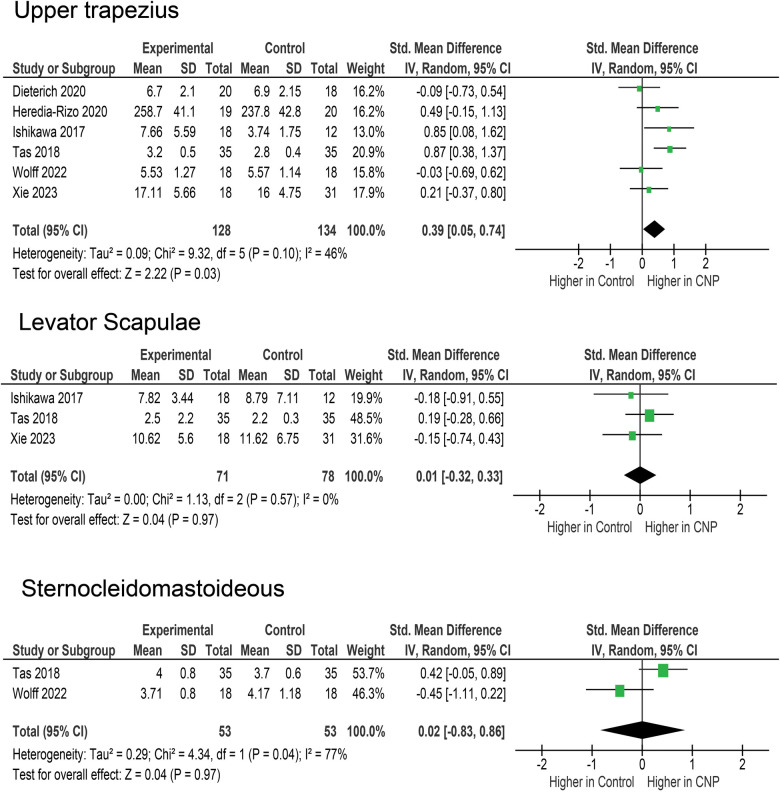
Meta-analyses for upper trapezius, levator scapulae and sternocleidomastoid.

Upper trapezius stiffness was assessed in 6 studies (Figure 2), with a total of 157 participants in CNP groups and 163 in control groups. The pooled difference (SMD = 0.39, 95% CI = 0.05–0.74) was statistically significant (*p* = 0.03). The heterogeneity between the studies was moderate (*I*^2^ = 46%). However, the sensitivity analysis showed that the pooled effect was relatively robust, ranging from 0.25 to 0.49 (*p* = 0.003–0.040) when studies were removed one-by-one.

Levator scapulae was assessed in three studies (21, 22, 42), with a total of 71 participants in CNP groups and 78 in control groups (Figure 2). The pooled effect did not indicate any differences between CNP and control groups (SMD = 0.01; 95% CI = −0.32–0.33; *p* = 0.970), and the effects were very homogenous across the studies (*I*^2^ = 0%).

Sternocleidomastoideus was assessed in two studies (40, 42), with a total of 53 participants in CNP groups and 53 in control groups (Figure 2). The pooled effect did not indicate any differences between CNP and control groups (SMD = 0.02; 95% CI = −0.83–0.86; *p* = 0.970). The heterogeneity between the studies was substantial (*I*^2^ = 77%).

**Figure 3:**
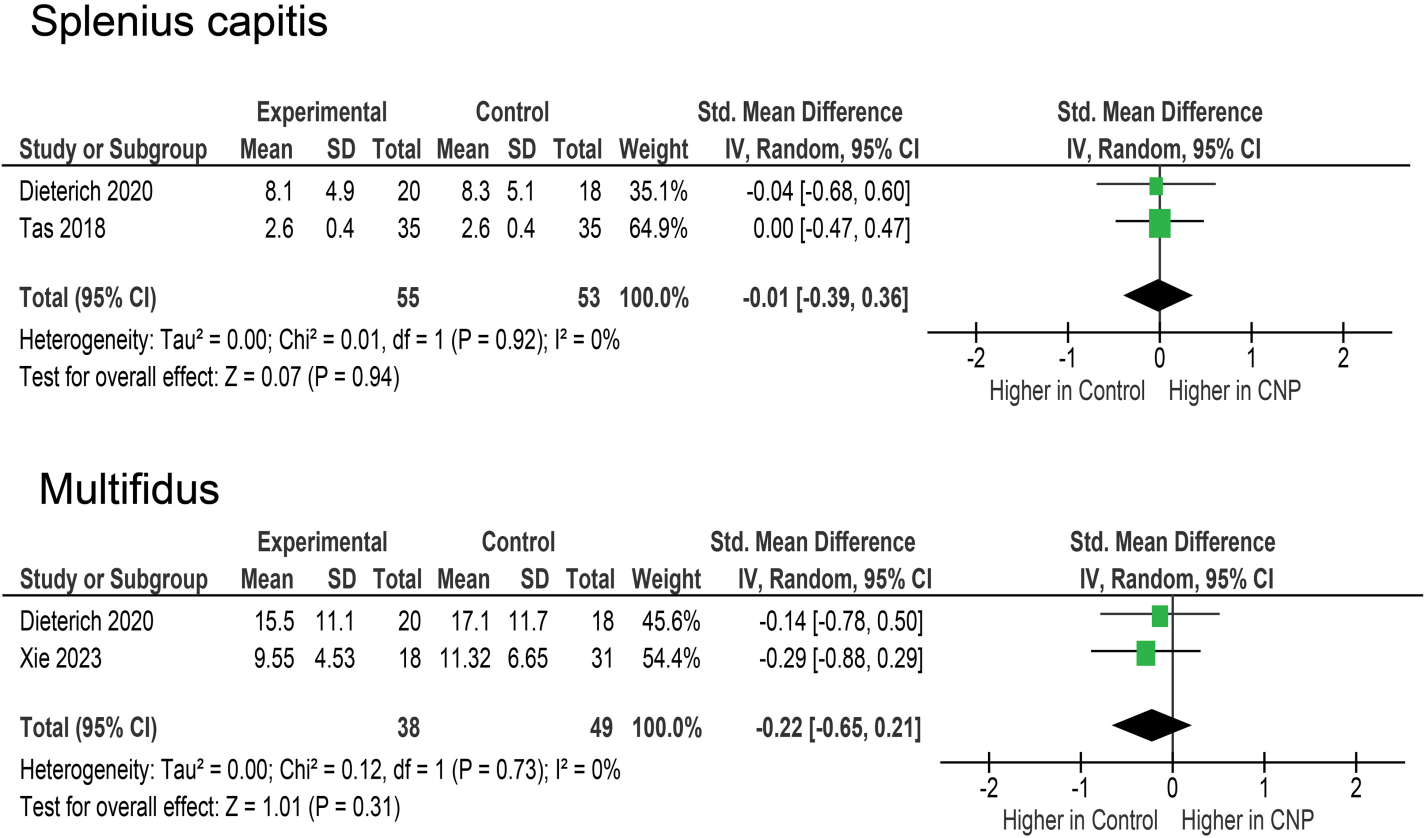
Meta-analyses for splenius capitis and multifidus.

Splenius capitis was assessed in two studies (21, 36), with a total of 71 participants in CNP groups and 78 in control groups (Figure 3). There were no differences between the CNP and control groups (SMD = −0.01; 95% CI = −0.39–0.36; *p* = 0.940) and the two studies very homogenous (*I*^2^ = 0%). Finally, multifidus was assessed in two studies (36, 42), with a total of 38 participants in CNP groups and 49 in control groups (Figure 3). Again, the pooled effect did not indicate a difference between the two groups (SMD = −0.22; 95% CI = −0.65–0.21; *p* = 0.310) and the two studies were very homogenous (*I*^2^ = 0%).

## 4. Discussion

The meta-analysis revealed a slight elevation in trapezius muscle stiffness among CNP patients in comparison to those without symptoms. Stiffness levels of the levator scapulae, splenius capitis, SCM, and multifidus appeared comparable between CNP patients and controls. It it crucial to emphasize that this systematic review, based on case-control studies, does not clarify whether the enhanced trapezius stiffness is a result of, or a reason for, CNP. However, further research is encouraged to elucidate if addressing trapezius stiffness might be an integral objective in CNP rehabilitation strategies.

Several studies, though they didn’t meet the criteria for inclusion in our meta-analysis, are worth mentioning for context. One study (43) analyzed the stiffness of upper trapezius and pectoralis minor by shear wave elastography in 39 female patients with rounded shoulder posture and neck pain. This study identified a significant positive correlation between the stiffness of the left upper trapezius and the shoulder angle in the rounded shoulder position (*r* = 0.44; *p* = 0.003). However, there was no observed relationship between the stiffness of the right upper trapezius, bilateral pectoralis minor muscles, and the angles of the shoulder and neck. A limitation of this study was the undefined duration of symptoms. The second study (44) assessed neck muscle stiffness in 20 participants, 3 of which had chronic neck pain and other 17 were healthy. Findings indicated a markedly higher stiffness in the trapezius muscle for those with chronic neck pain (*p* = 0.008) and a modest correlation between trapezius stiffness and body mass index (*r* = 0.34; *p* = 0.034). Yet, this study lacked clarity on the symptom duration for the CNP group. Kocur et al. (35) found increased stiffness in both upper trapezius and SCM, but failed to report the duration of the symptoms in their patients. Likewise, Onda et al. (34) reported increased stiffness in upper trapezius, but did not report the duration of the symptoms. Moreover, the meta-analysis could not be conducted for two muscles that were the focus of just a single study. Specifically, no significant difference was found between groups in the study of semispinalis cervicis and semispinalis capitis (36), with no difference between the groups. One study (22) also assessed rhomboid major, and also reported no differences between the groups.

The underlying mechanisms of increased trapezius stiffness in CNP patients are difficult to determine. It is plausible that pain and inflammation activate type III and IV sensory endings. This could stimulate gamma-motoneurons, potentially altering muscle spindle excitability, leading to increased muscle tone (45). Notably, most included studies [with one exception (40)] assessed passive muscle stiffness at rest (without voluntary contraction), examined passive muscle stiffness during rest, without any voluntary contraction. This raises questions about whether the observed stiffness in CNP patients indicates enhanced muscle activity at rest (26). There is prior evidence pointing to low-amplitude activity in the trapezius as a risk factor for neck and shoulder pain onset (46, 47). Alternatively, this augmented resting activation might act as a protective strategy to limit painful neck movements. This line of thought mirrors the hypotheses proposed for the observed increased stiffness in trunk extensors amongst low back pain patients (48). However, this does not fully account for why only the trapezius (and possibly SCM) displays increased stiffness. Additionally, stiffness measurements can vary based on muscle length (49), and thus on joint position. Poor neck posture may increase tension in the SCM muscle (50), which could be one of the risk factors for CNP, but also a confounding methodological factor when neck posture is not standardized in the assessment of stiffness. It would be beneficial for upcoming research on CNP patients to integrate EMG with shear-wave elastography. This approach could discern whether the increased stiffness is attributed to passive mechanical characteristics or elevated muscle activity. Such a distinction could profoundly impact treatment decisions. For instance, if addressing muscle mechanical properties is the goal, specific interventions like prolonged static stretching might be required (51).

While the exact role of stiffness in the trapezius in the onset of CNP remains uncertain, prioritizing the reduction of this stiffness could be a central objective of rehabilitation programs. In line with this, neck muscle stretching, especially of the trapezius, has been associated with immediate relief from neck pain (52). This is corroborated by a study which noted a simultaneous decrease in pain scores and upper trapezius muscle stiffness, as measured by elastography, in patients afflicted with myofascial pain syndrome. Contrary to our initial assumptions, we observed no heightened stiffness in the levator scapulae among CNP patients. Only one research (21) indicated a significant difference between CNP patients and control subjects, though the effect size was relatively small (specific study SMD = 0.42). This is particularly intriguing since the levator scapulae is frequently reported as tender among adults suffering from nonspecific neck pain (53). A potential limitation of the included studies is the lack of control for myofascial trigger points, which are also associated with increased elastography-based stiffness scores (54). Such active trigger points appear more frequently in the upper trapezius (40%) compared to the levator scapulae (15%) in those with mechanical neck pain (55). It is plausible that the observed trapezius stiffness in CNP could be attributed to the presence of myofascial trigger points. Consequently, stretching might alleviate pain by targeting these trigger points rather than purely addressing stiffness. This theory gains traction when considering research that has observed pain mitigation following stretching or other “release” techniques aimed at neck trigger points (56, 57). Irrespective of the exact mechanism at play, trapezius stretching may be a pivotal component in CNP treatment.

As we expected, no difference in the stiffness of other muscles (splenius capitis and multifidus) was found between the CNP and control groups. However, these results are based on a very limited number of studies (2 studies for each muscle); therefore, further studies are needed to reach a more solid conclusion. Multifidus stiffness tended to be even lower in CNP patients, suggesting that this muscle may benefit from strengthening in this group. Along with the transversus abdominis, the multifidus is often considered critical for healthy spinal function (58), and structural changes (e.g., atrophy and fat infiltration) in the multifidus muscle at the lumbar level have already been demonstrated in patients with low back pain (59). However, multifidus stiffness is increased in low back pain (60), which is contrary to the trends we have observed for the multifidus in CNP. This discrepancy is difficult to explain because different factors such as the decrease in muscle function and protective co-contraction could have opposite effects on the stiffness results. Nevertheless, in CNP, it might be justified to perform strengthening or stabilization exercises in addition to stretching exercises. Indeed, it has been shown that neck strengthening (61) and stabilization (62) may be beneficial in CNP patients, which could be related to improved multifidus function.

This systematic review does come with certain limitations that merit attention. Firstly, our conclusions are drawn strictly from case-control studies, meaning we cannot infer direct cause-and-effect relationships. For clarity on whether muscle stiffness is a precursor or an outcome of CNP, future research should adopt a prospective approach. Even though the case-control studies we included were generally of commendable quality, they employed varied assessment techniques (spanning elastography to myotonometry) and had participants with differing durations of symptoms. Notably, three of these studies were solely focused on women, while others incorporated both genders without conducting gender-specific analyses. Assessment of muscle stiffness may be influenced by factors such as adipose tissue content (63), which could cofound the relationship between stiffness scores and pain. Such discrepancies are especially significant for myotonometry, which concentrates on the muscle's superficial portions. However, the findings from myotonometry-centric studies generally mirrored those from elastography-centric ones, with no notable statistical discrepancies. Further reasons for heterogeneity include different elastography methods (strain elastography and shear-wave elastography) and different stiffness units (strain rate, shear modulus, shear-wave velocity). Yet, the limited number of studies at our disposal did not highlight any consistent effect of the assessment tool or outcome units on the results. Upcoming research should contemplate incorporating moderator variables, including age, sex, and body composition. Lastly, our literature exploration was constrained to three databases, possibly leading to the omission of some pertinent studies. Nevertheless, additional non-systematic searches in Google Scholar and reviews of reference lists uncovered 53 potential articles in our search's preliminary stage. However, none of these additional articles were integrated into the final review. This suggests that the search strategy was sufficient to include all relevant studies.

## 5. Conclusion

This article aimed to investigate which muscles exhibit increased stiffness in patients with CNP. Only a small increase in stiffness for the trapezius muscle was confirmed, whereas stiffness of the other muscles was similar in CNP patients and asymptomatic individuals. Nonetheless, a definitive link between muscle stiffness and CNP remains unestablished. As such, recommending a reduction in trapezius muscle stiffness as a primary rehabilitation strategy is still inconclusive and further research is needed.

## Data Availability

The original contributions presented in the study are included in the article/**Supplementary Material**, further inquiries can be directed to the corresponding author.

## References

[1] Cagnie B Danneels L Van Tiggelen D De Loose V Cambier D. Individual and work related risk factors for neck pain among office workers: a cross sectional study. Eur Spine J. (2007) 16:679–86. 10.1007/s00586-006-0269-7 17160393 PMC2213555

[2] Ostergren P-O. Incidence of shoulder and neck pain in a working population: effect modification between mechanical and psychosocial exposures at work? Results from a one year follow up of the Malmo shoulder and neck study cohort. J Epidemiol Community Heal. (2005) 59:721–8. 10.1136/jech.2005.034801 PMC1733134 16100307

[3] Tsakitzidis G Remmen R Dankaerts W Van Royen P. Non-specific neck pain and evidence-based practice. Eur Sci J. (2013) 9:1–19.

[4] Côté P Cassidy DJ Carroll LJ Kristman V. The annual incidence and course of neck pain in the general population: a population-based cohort study. Pain. (2004) 112:267–73. 10.1016/j.pain.2004.09.004 15561381

[5] Fejer R Kyvik KO Hartvigsen J. The prevalence of neck pain in the world population: a systematic critical review of the literature. Eur Spine J. (2006) 15:834–48. 10.1007/s00586-004-0864-4 15999284 PMC3489448

[6] Kang T Kim B. Cervical and scapula-focused resistance exercise program versus trapezius massage in patients with chronic neck pain: a randomized controlled trial. Medicine. (2022) 101:e30887. 10.1097/MD.0000000000030887 36181044 PMC9524908

[7] Falla D O’Leary S Farina D Jull G. The change in deep cervical flexor activity after training is associated with the degree of pain reduction in patients with chronic neck pain. Clin J Pain. (2012) 28:628–34. 10.1097/AJP.0b013e31823e9378 22156825

[8] Kristjansson E Treleaven J. Sensorimotor function and dizziness in neck pain: implications for assessment and management. J Orthop Sport Phys Ther. (2009) 39:364–77. 10.2519/jospt.2009.2834 19411769

[9] Williamson E Williams MA Gates S Lamb SE. Risk factors for chronic disability in a cohort of patients with acute whiplash associated disorders seeking physiotherapy treatment for persisting symptoms. Physiother. (2015) 101:34–43. 10.1016/j.physio.2014.04.004 24996567

[10] Yalcinkaya H Ucok K Ulasli AM Coban NF Aydin S Kaya I Do male and female patients with chronic neck pain really have different health-related physical fitness, depression, anxiety and quality of life parameters? Int J Rheum Dis. (2017) 20:1079–87. 10.1111/1756-185X.12389 24810182

[11] Page P Clare F Lardner R. Assessment and treatment of muscle imblance: the Janda approach. Champaign, Illinois, USA: Human Kinetics (2010).

[12] Johansson H Sojka P. Pathophysiological mechanisms involved in genesis and spread of muscular tension in occupational muscle pain and in chronic musculoskeletal pain syndromes: a hypothesis. Med Hypotheses. (1991) 35:196–203. 10.1016/0306-9877(91)90233-O 1943863

[13] MacDermid JC Walton DM Bobos P Lomotan M Carlesso L. A qualitative description of chronic neck pain has implications for outcome assessment and classification. Open Orthop J. (2016) 10:746–56. 10.2174/1874325001610010746 28217199 PMC5301418

[14] Falla DL Jull GA Hodges PW. Patients with neck pain demonstrate reduced electromyographic activity of the deep cervical flexor muscles during performance of the craniocervical flexion test. Spine. (2004) 29:2108–14. 10.1097/01.brs.0000141170.89317.0e 15454700

[15] Kim JY Kwag KI. Clinical effects of deep cervical flexor muscle activation in patients with chronic neck pain. J Phys Ther Sci. (2016) 28:269–73. 10.1589/jpts.28.269 26957772 PMC4756018

[16] Miranda IF Wagner Neto ES Dhein W Brodt GA Loss JF. Individuals with chronic neck pain have lower neck strength than healthy controls: a systematic review with meta-analysis. J Manipulative Physiol Ther. (2019) 42:608–22. 10.1016/j.jmpt.2018.12.008 31771837

[17] Wermeling M Scherer M Himmel W. GPs’ experiences of managing non-specific neck pain–a qualitative study. Fam Pract. (2011) 28:300–6. 10.1093/fampra/cmq109 21177744

[18] Simons GD Mense S. Understanding and measurement of muscle tone as related to clinical muscle pain. Pain. (1998) 75:1–17. 10.1016/S0304-3959(97)00102-4 9539669

[19] Ingram LA Rivett DA Snodgrass SJ. Comparison of cervical spine stiffness in individuals with chronic nonspecific neck pain and asymptomatic individuals. J Orthop Sports Phys Ther. (2015) 45:162–9. 10.2519/jospt.2015.5711 25627153

[20] Ranasinghe P Perera YS Lamabadusuriya DA Kulatunga S Jayawardana N Rajapakse S Work related complaints of neck, shoulder and arm among computer office workers: a cross-sectional evaluation of prevalence and risk factors in a developing country. Environ Heal A Glob Access Sci Source. (2011) 10. 10.1186/1476-069X-10-70 PMC3162880 21816073

[21] Taş S Korkusuz F Erden Z. Neck muscle stiffness in participants with and without chronic neck pain: a shear-wave elastography study. J Manipulative Physiol Ther. (2018) 41:580–8. 10.1016/j.jmpt.2018.01.007 30442356

[22] Ishikawa H Muraki T Morise S Sekiguchi Y Yamamoto N Itoi E Changes in stiffness of the dorsal scapular muscles before and after computer work: a comparison between individuals with and without neck and shoulder complaints. Eur J Appl Physiol. (2017) 117:179–87. 10.1007/s00421-016-3510-z 27913925

[23] Drakonaki EE Allen GM Wilson DJ. Ultrasound elastography for musculoskeletal applications. Br J Radiol. (2012) 85:1435–45. 10.1259/bjr/93042867 23091287 PMC3500785

[24] Blank J Blomquist M Arant L Cone S Roth J. Characterizing musculoskeletal tissue mechanics based on shear wave propagation - a 1 systematic review of current methods and reported measurements. Ann Biomed Eng. (2021) 50:751–68. 10.1007/s10439-022-02935-y PMC9631468 35359250

[25] Taljanovic MS Gimber LH Becker GW Latt LD Klauser AS Melville DM Shear-wave elastography: basic physics and musculoskeletal applications. Radiographics. (2017) 37:855–70. 10.1148/rg.2017160116 28493799 PMC5452887

[26] Kozinc Ž Šarabon N. Shear-wave elastography for assessment of trapezius muscle stiffness: reliability and association with low-level muscle activity. PLoS One. (2020) 15:e0234359. 10.1371/journal.pone.0234359 32520959 PMC7286494

[27] Sawada T Okawara H Nakashima D Iwabuchi S Matsumoto M Nakamura M Reliability of trapezius muscle hardness measurement: a comparison between portable muscle hardness meter and ultrasound strain elastography. Sensors. (2020) 20:1–10. 10.3390/s20247200 PMC7765603 33339151

[28] Ewertsen C Carlsen J Perveez MA Schytz H. Reference values for shear wave elastography of neck and shoulder muscles in healthy individuals. Ultrasound Int Open. (2018) 4:E23–9. 10.1055/s-0044-102013 29629427 PMC5886310

[29] Young BA Koppenhaver SL Timo-Dondoyano RM Baumann K Scheirer VF Wolff A Ultrasound shear wave elastography measurement of the deep posterior cervical muscles: reliability and ability to differentiate between muscle contraction states. J Electromyogr Kinesiol. (2021) 56:102488. 10.1016/j.jelekin.2020.102488 33189075

[30] McGowen JM Hoppes CW Forsse JS Albin SR Abt J Koppenhaver SL. The utility of myotonometry in musculoskeletal rehabilitation and human performance programming. J Athl Train. (2022). 10.4085/1062-6050-0616.21. [Epub ahead of print] 37418563 PMC11215642

[31] Voglar M Vatovec R Kozinc Ž Šarabon N. The effects of eccentric exercise on passive hamstring muscle stiffness: comparison of shear-wave elastography and passive knee torque outcomes. Eur J Transl Myol. (2022) 32:10567. 10.4081/ejtm.2022.10567 35666465 PMC9295161

[32] Kisilewicz A Madeleine P Ignasiak Z Ciszek B Kawczynski A Larsen RG. Eccentric exercise reduces upper trapezius muscle stiffness assessed by shear wave elastography and myotonometry. Front Bioeng Biotechnol. (2020) 8:928. 10.3389/fbioe.2020.00928 32903634 PMC7438744

[33] Garcia-Bernal MI Heredia-Rizo AM Gonzalez-Garcia P Cortés-Vega MD Casuso-Holgado MJ. Validity and reliability of myotonometry for assessing muscle viscoelastic properties in patients with stroke: a systematic review and meta-analysis. Sci Rep. (2021) 11:5062. 10.1038/s41598-021-84656-1 33658623 PMC7930253

[34] Onda A Onozato K Kimura M. Clinical features of neck and shoulder pain (Katakori) in Japanese hospital workers. Fukushima J Med Sci. (2022) 68:79–87. 10.5387/fms.2022-02 35660659 PMC9493333

[35] Kocur P Wilski M Lewandowski J Łochyński D. Female office workers with moderate neck pain have increased anterior positioning of the cervical spine and stiffness of upper trapezius myofascial tissue in sitting posture. PMR. (2019) 11:476–82. 10.1016/j.pmrj.2018.07.002 31034771

[36] Dieterich AV Yavuz UŞ Petzke F Nordez A Falla D. Neck muscle stiffness measured with shear wave elastography in women with chronic nonspecific neck pain. J Orthop Sports Phys Ther. (2020) 50:179–88. 10.2519/jospt.2020.8821 31905095

[37] Page MJ McKenzie JE Bossuyt PM Boutron I Hoffmann TC Mulrow CD The PRISMA 2020 statement: an updated guideline for reporting systematic reviews. Br Med J. (2021) 88:n71. 10.1136/bmj.n71 PMC8005924 33782057

[38] Moskalewicz A Oremus M. No clear choice between Newcastle–Ottawa scale and appraisal tool for cross-sectional studies to assess methodological quality in cross-sectional studies of health-related quality of life and breast cancer. J Clin Epidemiol. (2020) 120:94–103. 10.1016/j.jclinepi.2019.12.013 31866469

[39] Borenstein M Hedges L Higgins J Rothstein H. Introduction to meta-analysis. Hoboken, New Yersey, USA: John Wiley & Sons (2021).

[40] Wolff WL Heinemann CM Lipps DB. The influence of idiopathic chronic neck pain on upper trapezius and sternocleidomastoid muscle activity and elasticity during functional reaching: a cross-sectional study. J Biomech. (2022) 141:111223. 10.1016/j.jbiomech.2022.111223 35926366

[41] Heredia-Rizo AM Petersen KK Arendt-Nielsen L Madeleine P. Eccentric training changes the pressure pain and stiffness maps of the upper trapezius in females with chronic neck-shoulder pain: a preliminary study. Pain Med. (2020) 21:1936–46. 10.1093/PM/PNZ360 32011710

[42] Xie Y Thomas L Johnston V Coombes BK. Cervical and axioscapular muscle stiffness measured with shear wave elastography: a comparison between different levels of work-related neck disability. J Electromyogr Kinesiol. (2023) 69:102754. 10.1016/j.jelekin.2023.102754 36773478

[43] Ertekin E Günaydın ÖE. Neck pain in rounded shoulder posture: clinico-radiologic correlation by shear wave elastography. Int J Clin Pract. (2021) 75:e14240. 10.1111/ijcp.14240 33971068

[44] Kuo WH Jian DW Wang TG Wang YC. Neck muscle stiffness quantified by sonoelastography is correlated with body mass index and chronic neck pain symptoms. Ultrasound Med Biol. (2013) 39:1356–61. 10.1016/j.ultrasmedbio.2012.11.015 23683408

[45] Knutson GA. The role of the gamma-motor system in increasing muscle tone and muscle pain syndromes: a review of the Johansson/Sojka hypothesis. J Manipulative Physiol Ther. (2000) 23:564. 10.1067/mmt.2000.109674 11050614

[46] Mork PJ Westgaard RH. Low-amplitude trapezius activity in work and leisure and the relation to shoulder and neck pain. J Appl Physiol. (2006) 100:1142–9. 10.1152/japplphysiol.01111.2005 16322372

[47] Westgaard RH Vasseljen O Holte KA. Trapezius muscle activity as a risk indicator for shoulder and neck pain in female service workers with low biomechanical exposure. Ergonomics. (2001) 44:339–53. 10.1080/00140130119649 11219764

[48] Koppenhaver S Gaffney E Oates A Eberle L Young B Hebert J Lumbar muscle stiffness is different in individuals with low back pain than asymptomatic controls and is associated with pain and disability, but not common physical examination findings. Musculoskelet Sci Pract. (2020) 45:102078. 10.1016/j.msksp.2019.102078 31704551

[49] Lacourpaille L Nordez A Hug F Doguet V Andrade R Guilhem G. Early detection of exercise-induced muscle damage using elastography. Eur J Appl Physiol. (2017) 117:2047–56. 10.1007/s00421-017-3695-9 28780603

[50] Rubine-Gatina S Rimere N Zundane Z Gulajeva A Reste J. Sternocleidomastoid muscle and head position: how to minimize muscle tension. IISE Trans Occup Ergon Hum Factors. (2022) 10:192–200. 10.1080/24725838.2022.2141369 36308294

[51] Takeuchi K Nakamura M Konrad A Mizuno T. Long-term static stretching can decrease muscle stiffness: a systematic review and meta-analysis. Scand J Med Sci Sports. (2023) 33:1294–306. 10.1111/sms.14402 37231582

[52] Ylinen J Kautiainen H Wirén K Häkkinen A. Stretching exercises vs manual therapy in treatment of chronic neck pain: a randomized, controlled cross-over trial. J Rehabil Med. (2007) 39:126–32. 10.2340/16501977-0015 17351694

[53] Andersen LL Hansen K Mortensen OS Zebis MK. Prevalence and anatomical location of muscle tenderness in adults with nonspecific neck/shoulder pain. BMC Musculoskelet Disord. (2011) 12:169. 10.1186/1471-2474-12-169 21777478 PMC3161919

[54] Lok U Huang C Zhou C Yang L Ling W Tang S Quantitative shear wave speed assessment for muscles with the diagnosis of taut bands and/or myofascial trigger points using probe oscillation shear wave elastography: a pilot study. J Ultrasound Med. (2022) 41:845–54. 10.1002/jum.15764 34085301 PMC8642490

[55] Muñoz-Muñoz S Muñoz-García MT Alburquerque-Sendín F Arroyo-Morales M Fernández-De-Las-Peñas C. Myofascial trigger points, pain, disability, and sleep quality in individuals with mechanical neck pain. J Manipulative Physiol Ther. (2012) 35:608–13. 10.1016/j.jmpt.2012.09.003 23158466

[56] Wilke J Vogt L Niederer D Hübscher M Rothmayr J Ivkovic D Short-term effects of acupuncture and stretching on myofascial trigger point pain of the neck: a blinded, placebo-controlled RCT. Complement Ther Med. (2014) 22:835–41. 10.1016/j.ctim.2014.09.001 25440373

[57] Jaeger B Reeves JL. Quantification of changes in myofascial trigger point sensitivity with the pressure algometer following passive stretch. Pain. (1986) 27:203–10. 10.1016/0304-3959(86)90211-3 3797015

[58] Wong AYL Parent EC Funabashi M Stanton TR Kawchuk GN. Do various baseline characteristics of transversus abdominis and lumbar multifidus predict clinical outcomes in nonspecific low back pain? A systematic review. Pain. (2013) 154:2589–602. 10.1016/j.pain.2013.07.010 23867731

[59] Goubert D van Oosterwijck J Meeus M Danneels L. Structural changes of lumbar muscles in non-specific low back pain. Pain Physician. (2016) 19:E985–1000. 10.36076/ppj/2016.19.e985 27676689

[60] Murillo C Falla D Sanderson A Rushton A Heneghan NR. Shear wave elastography investigation of multifidus stiffness in individuals with low back pain. J Electromyogr Kinesiol. (2019) 47:19–24. 10.1016/j.jelekin.2019.05.004 31077992

[61] Salo PK Häkkinen AH Kautiainen H Ylinen JJ. Effect of neck strength training on health-related quality of life in females with chronic neck pain: a randomized controlled 1-year follow-up study. Health Qual Life Outcomes. (2010) 8:48. 10.1186/1477-7525-8-48 20465854 PMC2877013

[62] Celenay ST Kaya DO Akbayrak T. Cervical and scapulothoracic stabilization exercises with and without connective tissue massage for chronic mechanical neck pain: a prospective, randomised controlled trial. Man Ther. (2016) 21:144–50. 10.1016/j.math.2015.07.003 26211422

[63] Yoshiko A Ando R Akima H. Passive muscle stiffness is correlated with the intramuscular adipose tissue in young individuals. Eur J Appl Physiol. (2023). 123:1081–90. 10.1007/s00421-023-05137-z 36637509

